# A Novel Nomogram for Predicting Survival in Patients with Severe Acute Pancreatitis: An Analysis Based on the Large MIMIC-III Clinical Database

**DOI:** 10.1155/2021/9190908

**Published:** 2021-10-11

**Authors:** Didi Han, Fengshuo Xu, Chengzhuo Li, Luming Zhang, Rui Yang, Shuai Zheng, Zichen Wang, Jun Lyu

**Affiliations:** ^1^Department of Clinical Research, The First Affiliated Hospital of Jinan University, Guangzhou 510630, Guangdong Province, China; ^2^School of Public Health, Xi'an Jiaotong University Health Science Center, Xi'an 710061, Shaanxi Province, China; ^3^Intensive Care Unit, The First Affiliated Hospital of Jinan University, Guangzhou 510630, China; ^4^School of Public Health, Shannxi University of Chinese Medicine, Xianyang, Shaanxi, China; ^5^Department of Public Health, University of California, Irvine 92697, California, USA

## Abstract

**Background:**

Severe acute pancreatitis (SAP) can cause various complications. Septic shock is a relatively common and serious complication that causes uncontrolled systemic inflammatory response syndrome, which is one of the main causes of death. This study aimed to develop a nomogram for predicting the overall survival of SAP patients during the initial 24 hours following admission.

**Materials and Methods:**

All the data utilized in this study were obtained from the MIMIC-III (Medical Information Mart for Intensive Care III) database. The data were analyzed using multivariate Cox regression, and the performance of the proposed nomogram was evaluated based on Harrell's concordance index (C-index) and the area under the receiver operating characteristic curve (AUC). The clinical value of the prediction model was tested using decision-curve analysis (DCA). The primary outcomes were 28-day, 60-day, and 90-day mortality rates.

**Results:**

The 850 patients included in the analysis comprised 595 in the training cohort and 255 in the validation cohort. The training cohort consisted of 353 (59.3%) males and 242 (40.7%) females with SAP. Multivariate Cox regression showed that weight, sex, insurance status, explicit sepsis, SAPSII score, Elixhauser score, bilirubin, anion gap, creatinine, hematocrit, hemoglobin, RDW, SPO_2_, and respiratory rate were independent prognostic factors for the survival of SAP patients admitted to an intensive care unit. The predicted values were compared using C-indexes, calibration plots, integrated discrimination improvement, net reclassification improvement, and DCA.

**Conclusions:**

We have identified some important demographic and laboratory parameters related to the prognosis of patients with SAP and have used them to establish a more accurate and convenient nomogram for evaluating their 28-day, 60-day, and 90-day mortality rates.

## 1. Background

Acute pancreatitis (AP) is a clinically common condition that is characterized by sudden inflammation of the pancreas and the release of a series of digestive enzymes, leading to local tissue damage and multiple organ dysfunction syndrome (MODS) [1]. It is a critical disease with a high fatality rate, and especially in severe acute pancreatitis (SAP) the tissues release various factors including activated trypsin, a large number of cytokines, and inflammatory mediators, which aggravate the inflammatory necrosis of the pancreas and can even involve lesions in adjacent organs and tissues. Approximately 25% of AP patients develop SAP, and the mortality rate has been reported to vary from 10% to 60% [2, 3]. SAP can cause various complications, among which septic shock is a relatively common and serious one, causing uncontrolled systemic inflammatory response syndrome, which is one of the main causes of death [4]. Early effective treatment can improve the prognosis of patients [5]. Literature reviews have found that factors such as age, RDW, WBC, albumin, ALT, MODS, and SPO_2_ are significantly associated with a higher risk of death in SAP patients [6, 7].

Effective diagnostic methods and optimal strategies for SAP have not yet been determined, which requires the development of a severity assessment model to stratify patients at risk [8]. Although the existing critical-care scoring tools are designed for clinical use, there is no effective and rapidly calculated bedside prognostic scoring tool for predicting the prognosis of SAP patients in general intensive care units (ICUs). Thus, knowledge of the prognostic factors for SAP may play a critical role in helping clinicians identify high-risk patients and make better medical judgments.

A nomogram is a convenient mathematical tool for predicting certain endpoints, such as disease progression or death [9], based on several various key parameters. It is used to calculate the probability of a clinical event based on the prognostic weights of multiple factors [10]. A nomogram is a powerful and easy-to-use clinical decision-maker that can predict the outcomes in individual patients, thus benefiting both patients and clinicians alike [11].

The main aim of this study was to integrate multiple independent risk factors of SAP. These risk factors come from the Medical Information Mart for Intensive Care III (MIMIC-III) database, and they were used to establish a prognostic nomogram for improving predictions of the overall survival of SAP patients.

We present the following article/case in accordance with the CONSORT reporting checklist.

## 2. Methods

### 2.1. Data Source

All data in this study were obtained from the MIMIC-III database, which includes approximately 60,000 pieces of unconfirmed health-related data from the ICU of the Beth Israel Deaconess Medical Center (BIDMC) in the United States [12, 13]. All of the subjects had been admitted to the ICU of BIDMC between 2001 and 2012 [14]. Researchers around the world can use the data in the MIMIC-III database for free as long as they pass the assessment established by this database and obtain the data-use protocol [15, 16].

We obtained permission to access the database after completing the online training course of the National Health Protection Human Research Institute (certification number: 38292153).

### 2.2. Patients and Inclusion Criteria

**Figure 1:**
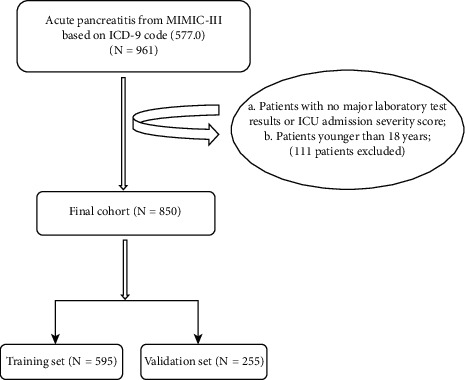
Study cohort. Illustration of selection criteria as utilized to select the final cohort of 850 patients.

Code 577.0 of the ICD-9 specification was used to identify 961 patients who were admitted to an ICU with AP for the first time [17]. We excluded patients with no major laboratory test results or ICU admission severity score within the first 24 hours and also patients below 18 years of age. Finally, 850 people who met the inclusion criteria were selected. The procedure of data selection according to the criteria mentioned above is presented in Figure 1.

We identified the all-cause death rates at 28 days, 60 days, and 90 days after ICU admission as the primary outcomes of our study. We randomly selected 70% of the eligible patients as the training cohort and used the remaining 30% of patients to independently validate the data.

### 2.3. Data Extraction and Management

Navicat Premium software was used to extract the raw data at 24 hours after ICU admission, and R software (version 3.6.1) was used for further data processing. This software supports MIMIC-III documents and other websites that are publicly available [18]. The code used to generate the descriptive statistics can be accessed at https://github.com/MIT-LCP/mimic-code/tree/master/concepts [12].

The data collected within 24 hours of ICU admission included demographics, vital signs (e.g., mean blood pressure, temperature, SPO_2_, respiratory rate, and heart rate), laboratory tests (e.g., RDW, chloride, glucose, sodium, and WBC), severity scores on scoring systems, and comorbidities. Demographic characteristics including age, sex, race, insurance status, marital status, readmission records, and survival time were collected from the original data set. The baseline characteristics, severity scores on scoring systems, and Elixhauser score of the patients were calculated as described previously [19–21]. The primary endpoints of this study were the mortality rates at 28 days, 60 days, and 90 days from the date of ICU admission.

### 2.4. Statistical Analysis

Continuous variables conforming to a normal distribution are presented as mean ± SD or median and interquartile-range (IQR) values, while categorical variables are presented as frequencies and proportions. Continuous variables were compared using the *t*-test or Wilcoxon rank-sum test, as appropriate, while categorical variables were compared using *χ*^2^ or Fisher's exact tests. Backward stepwise selection was applied in a Cox regression model to select variables in the training cohort. The prognostic factors that were identified as being significant were used to construct a nomogram for predicting the 28-day, 60-day, and 90-day survival rates of AP patients.

Harrell's concordance index (C-index) and the area under the receiver operating characteristic curve (AUC) were used to evaluate the predictive accuracy of the constructed nomogram. A calibration curve was used to evaluate the consistency between the predicted probabilities and the actual outcomes [15]. For the new prediction model, the net reclassification improvement (NRI) was used to compare the accuracy of two models, while the integrated discrimination improvement (IDI) was used to determine the effectiveness of the improvements. The clinical value of the prediction model was tested using decision-curve analysis (DCA).

We used R software (version 3.6.1, CRAN) and SPSS (version 24.0, Chicago, IL) for the statistical analyses. Probability values of *P* < 0.05 in two-sided tests were regarded as being statistically significant.

## 3. Results

### 3.1. Baseline Characteristics of Patients

The study included 850 eligible AP patients: 595 in the training cohort and 255 in the validation cohort. The training cohort consisted of 353 (59.3%) males and 242 (40.7%) females with AP with a median age of 60 years (IQR = 48–71 years). The validation cohort comprised 130 (51.0%) males and 125 (49.0%) females with a median age of 59 years (IQR = 45–71 years). Most of the patients in both cohorts were white (>65%), male (>50%), and married (>60%) and had Medicare (46.1%) or private (36.0%) insurance. Most patients did not have an infection or MODS.

Only the sex distribution and glucose differed significantly between the training and validation cohorts. The baseline clinicopathological data were similar in the training and validation cohorts, as indicated in Table 1. The median length of stay in the ICU was 6 days (IQR = 0–102 days). Long-term outcome data were available for all 850 patients: the 28-day, 60-day, and 90-day mortality rates were 12.9% (*n* = 110), 18.7% (*n* = 159), and 53.2% (*n* = 452), respectively.

### 3.2. Prognostic Factors for 28-Day, 60-Day, and 90-Day Mortality

Univariate analyses revealed that the significant variables were weight, sex, insurance status, explicit sepsis, SAPSII score, Elixhauser score, bilirubin, anion gap, creatinine, hematocrit, hemoglobin, RDW, SPO_2_, heart rate, and respiratory rate, which were included in multivariate Cox regression analyses. The multivariate analyses showed that the positive factors for survival were weight (HR = 0.989, *P*=0.001), being female (HR = 0.728 versus male, *P* < 0.05), having private insurance (HR = 0.496 versus Medicare, *P* < 0.01), having Medicaid (HR = 0.674 versus Medicare, *P* = 0.096), having government insurance (HR = 0.269 versus Medicare, *P* < 0.05), creatinine (HR = 0.820, *P* < 0.001), hemoglobin (HR = 0.678, *P* < 0.001), SPO_2_ (HR = 0.938, *P*=0.001), and heart rate (HR = 0.986 *P* < 0.001). In addition, the risk factors affecting survival were explicit sepsis (HR = 2.052 versus without explicit sepsis, *P* < 0.001), SAPSII score (HR = 1.037, *P* < 0.001), Elixhauser score (HR = 1.024, *P* = 0.002), bilirubin (HR = 1.043, *P*=0.010), anion gap (HR = 1.038, *P*=0.001), hematocrit (HR = 1.116, *P* = 0.001), RDW (HR = 1.148, *P* < 0.001), and respiratory rate (HR = 1.045, *P*=0.009).

### 3.3. Prognostic Nomogram for 28-Day, 60-Day and 90-Day Mortality

**Figure 2:**
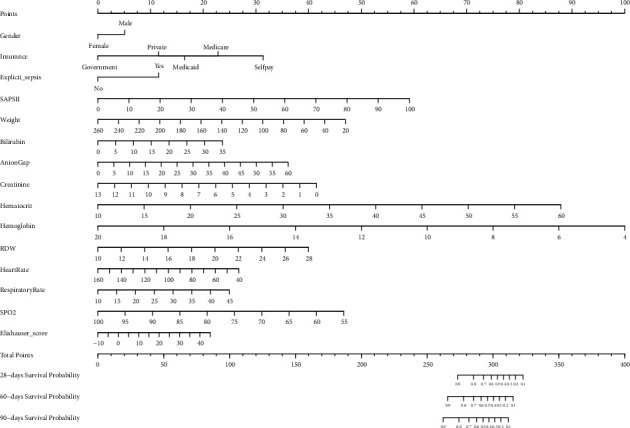
Nomogram predicting 28-, 60-, and 90-day mortality. The point of each variable was then summed up to obtain a total score that corresponds to a predicted probability of 28-, 60-, and 90-day death at the bottom of the nomogram.

The results of the multivariate regression model presented in Table 2 were used to establish a nomogram (Figure 2). The nomogram contained all of important independent factors predicting 28-day, 60-day, and 90-day mortality in the training cohort. The nomogram indicates that hemoglobin is the most important factor affecting prognosis, and it also includes sex, insurance status, explicit sepsis, Elixhauser score, weight, bilirubin, anion gap, creatinine, hematocrit, hemoglobin, RDW, heart rate, respiratory rate, and SPO_2_.

### 3.4. Performance and Clinical Usefulness of the Nomogram

**Figure 3:**
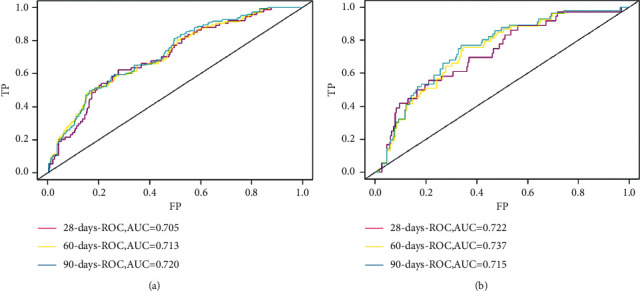
ROC curves. The ability of the model to be measured by the C-index. (a) Training cohort; (b) validation cohort. ROC: receiver operating characteristic.

**Figure 4:**
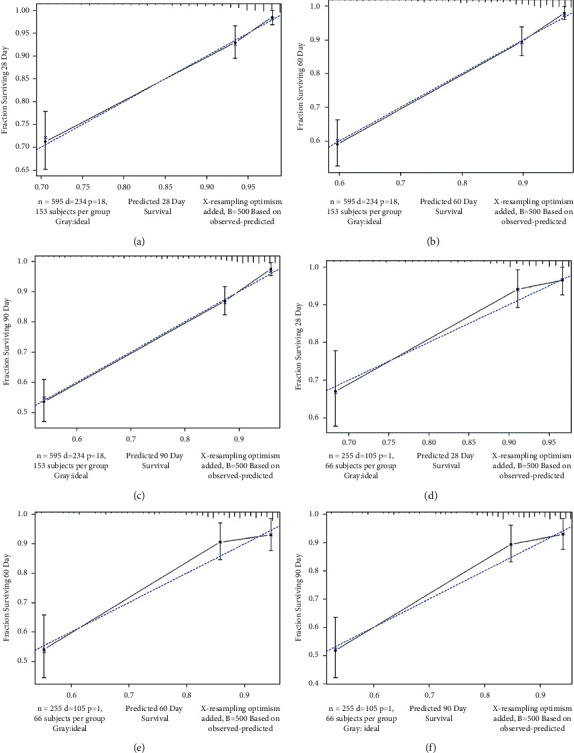
Calibration plots showing the relationship between the predicted probabilities based on the nomogram and actual values of the training cohort (a, b, c) and validation cohort (d, e, f).

**Figure 5:**
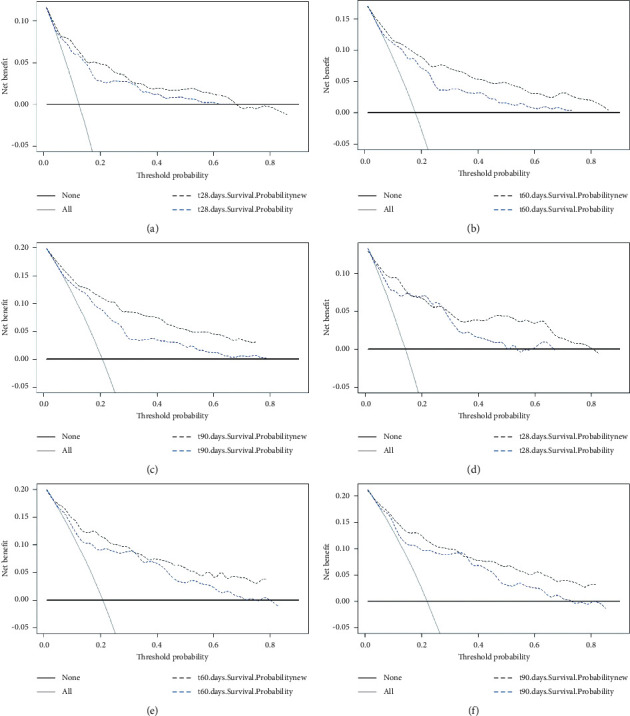
Decision-curve analysis. The abscissa is the threshold probability, and the ordinate is the net benefit rate. The horizontal one indicates that all samples are negative and all are not treated, with a net benefit of 0. The oblique one indicates that all samples are positive. The net benefit is a backslash with a negative slope. (a, b, c) Training cohort and (d, e, f) validation cohort.

The C-index analysis of the training cohort indicated that the nomogram provided high 28-day, 60-day, and 90-day survival C-indexes of 0.705, 0.713, and 0.720, respectively. The C-indexes of the nomogram were similarly high in the internal validation cohort, at 0.722, 0.737, and 0.751, indicating the good discriminative ability of the model (Figure 3). All of the C-indexes exceed 0.700, and the calibration curve has good consistency with the 45-degree ideal line (Figure 4). The DCA curves in Figure 5 display the large net benefits of the new model in predicting survival at 28 days, 60 days, and 90 days.

### 3.5. Predictive Accuracy of the Nomogram

The NRI values at the 28-day, 60-day, and 90-day follow-ups were 0.501, 0.704, and 0.732, respectively, in the training cohort and 0.170, 0.299, and 0.314, respectively, in the validation cohort. The results show that the new model has better prediction performance than the SAPSII model. Moreover, the IDI values at the 28-day, 60-day, and 90-day follow-ups were 0.084, 0.107, and 0.118, respectively, in the training cohort, and 0.041, 0.077, and 0.085, respectively, in the validation cohort.

## 4. Discussion

AP is a sudden inflammatory process of the pancreas with a mortality rate of 10%. The severity of AP ranges from a mild self-limiting disease to systemic complications and MODS. Up to 25% of SAP patients become critically ill, with a mortality rate of 20–30% [22, 23]. Moreover, the clinical symptoms vary with the body weight, with lighter people experiencing pancreas edema that manifests as abdominal pain, diarrhea, and vomiting, whereas in heavier people the pancreas exhibits necrosis, sepsis, or hemorrhage, resulting in shock and peritonitis, which is a dangerous condition with even higher mortality [24]. The onset age is between 20 years and 50 years, and it is more common in females than in males. Bleeding and necrosis are the most serious findings in AP, since they can readily cause peritonitis. Some patients will experience MODS or even sudden death.

The current clinical evaluation indicators for the severity and prognosis of acute pancreatitis are Ranson score [25] and SAPS score [26, 27]. Ranson score requires 48 h of inpatient observation, thereby resulting in delayed triage and management [28, 29]. The SAPSII score involves 17 clinical variables. The above two scoring systems have the characteristics of too many variables involved, being expensive, complex calculation process, and poor feasibility [30]. It is therefore necessary to establish a model for the prognosis that is simple to use and more accurate than the traditional SAP scoring system.

This study used the publicly available MIMIC-III database [12], which contains detailed information on 38,161 patients, including their vital signs, severity scores on scoring systems, laboratory tests, and diagnostic information and so can be used to obtain adequate clinical information about patients.

A nomogram is a prediction tool in the form of a simple chart based on statistical forecasting models. The prognostic weight of each factor is taken into account when calculating the probability of a particular clinical event [10]. The simplicity of the diagrammatic form of a nomogram additionally helps clinicians to provide patients and their families with effective consultations about risks, allowing them to make objective decisions. The present study established a nomogram for predicting the 28-day, 60-day, and 90-day mortality rates for AP based on the MIMIC-III database.

Our nomogram model is based on readily available clinical factors. We compared the prognostic model with the traditional APSIII scoring system using several commonly used model validation parameters: C-index, calibration curves, NRI, IDI, and DCA curve.

The multivariate Cox regression performed in this study showed that weight, sex, insurance status, explicit sepsis, SAPSII score, Elixhauser score, bilirubin, anion gap, creatinine, hematocrit, hemoglobin, RDW, SPO_2_, and respiratory rate are independent prognostic factors for the survival of SAP patients admitted to ICUs. The influence of overweight and obesity on the severity of AP has been controversial for a long time [31–34]. Interestingly, current research shows that greater weight reduces the risk of poor prognosis in AP patients. We believe that this may be due to the poorer physical condition of AP patients in the ICU, and patients with larger weight at the time of admission tend to have better physical nutritional status, so the long-term prognosis of patients is better. Sex differences in AP have been found in previous studies, with males being more likely to develop the disease and have a worse prognosis than females. Consistent with previous studies [35], the present study found that females have better survival rates compared to males.

A particularly interesting finding of the present research was the type of insurance for patients in the ICU also being a significant prognostic factor. Patients with private, Medicaid, or government medical insurance had a better prognosis than patients with only a self-pay plan. This is in line with patients with good insurance and financial conditions usually receiving better treatment and care and recovering more quickly.

The disease severity has a major influence on the mortality rate in AP [36], with this being higher in patients with SAP and comorbidities than in those with mild AP. This is consistent with the present study finding that SAPSII and Elixhauser scores are positively correlated with the mortality of AP patients. Moreover, patients with AP may develop sepsis as their condition worsens, and patients with concurrent sepsis often have a poor prognosis. Similarly, our study found that patients with significant sepsis were at an increased risk of poor outcomes.

Regarding laboratory indexes, we found that creatinine, hemoglobin, bilirubin, anion gap, hematocrit, RDW, and SPO_2_ were significant independent prognostic indicators. Previous studies have shown that serum creatinine level is a good predictor of AP, and persistently high serum creatinine levels indicate poor prognosis for AP patients [37, 38]. The reason why our research results are inconsistent with previous studies may be that, in the MIMIC-III database, the time to collect patient biochemical indicators was earlier time, and continuous monitoring results were not obtained, so it cannot accurately reflect the prognosis of long-term survival. Hemoglobin is another laboratory test used, which may reflect changes in the intravascular volume status and is taken into account in cases of cardiovascular dysfunction [39]. In our study, we found that a decrease in hemoglobin was correlated with increased mortality.

Our study suggests that elevated bilirubin levels are a risk factor for SAP. Previous studies [3] have found that when AP occurs, obstruction of the bile duct can block bile excretion, and bilirubin will be deposited in liver cells, which affects the normal metabolism of liver cells and leads to the degeneration and necrosis of liver cells and impaired liver function. This increases the bilirubin level and results in a poor prognosis. It has also been reported [40] that increased hematocrit is associated with pancreatic necrosis and organ failure. We similarly found that hematocrit is a risk factor for the prognosis in AP patients.

The relationships between the anion gap and the clinical outcomes of various diseases have been explored previously [41, 42]. The anion gap is a traditional tool for assessing the acid-base status, and most previous studies have linked it to acid-base disorders, which have a main impact on the morbidity and mortality of critically ill patients [43]. Similarly, we found that the serum anion gap is a risk factor for death in patients with SAP. RDW has traditionally been an important indicator in complete blood count testing, representing the variability of the red blood cell volume [44]. A recent systematic review [45] found that the admission RDW can be used as an independent biomarker of a high risk of death in AP patients. Our results support this conclusion. Also, a lower SPO_2_ contributing to a higher mortality rate is similar to previous findings [37].

The main strength of our study is that it has produced the first comprehensive nomogram for the prognosis of ICU patients with AP. The nomogram represents a further improvement over the traditional SAPSII score and includes more prognostic indicators. Nevertheless, our study also had several limitations. Firstly, we used data from a single center in the United States, which may have resulted in selection bias and thus limits the applicability of our findings to other regions. However, performing the study in a single center increased the likelihood of the patient treatments being more consistent. Secondly, we only collected the laboratory parameters that were measured when a patient entered the ICU, and there were no laboratory follow-up data. It is possible that measurement data were incorrectly classified, which may have affected the results of the study. Finally, because the data in the MIMIC-III database are relatively old, the database could only be used for internal validation. Therefore, in future research we need to conduct external verification based on our own data to further verify the performance and accuracy of the new nomogram [46].

## 5. Conclusions

This study identified important demographic and laboratory parameters related to the prognosis of patients with SAP and used this information to establish a more accurate and convenient nomogram for evaluating the prognosis. The new nomogram clearly shows the 28-day, 60-day, and 90-day all-cause mortality rates for SAP patients in ICUs. The prognostic value of the single SAPSII scoring system was found to be inferior to that of the novel nomogram. Applying our new nomogram in the clinical care environment can help doctors who are making treatment and management decisions about SAP. However, larger prospective studies with longer follow-up times are needed to further confirm the present findings.

## Figures and Tables

**Table 1:** Baseline characteristics, vital signs, laboratory parameters, and outcomes of patients with acute pancreatitis.

Variables	Classification	Patients		*P*
		Training set (%)	Validation set (%)	
Total	—	595	255	—
Age	—	60 (48–71)	59 (45–71)	0.450
LOS	—	4 (2–12)	3 (2–10)	0.128
Weight	—	81 (68–97)	79 (68–91)	0.145

Gender	Male	353 (59.3)	130 (51.0)	0.024
	Female	242 (40.7)	125 (49.0)	

Ethnicity	White	400 (67.2)	168 (65.9)	0.790
	Black	58 (9.7)	23 (9.0)	
	Other	137 (23.0)	64 (25.1)	

Admission type	Elective	15 (2.5)	11 (4.3)	0.204
	Emergence	557 (93.6)	230 (90.2)	
	Urgent	23 (3.9)	14 (5.5)	

Insurance	Medicare	274 (46.1)	116 (45.5)	0.908
	Private	214 (36.0)	95 (37.3)	
	Medicaid	73 (12.3)	33 (12.9)	
	Government	27 (4.5)	8 (3.1)	
	Self-pay	7 (1.2)	3 (1.2)	

Marital status	Married	366 (61.5)	166 (65.1)	0.580
	Unmarried	184 (30.9)	73 (28.6)	
	Other	45 (7.6)	16 (6.3)	

Explicit sepsis	Yes	470 (79.0)	198 (77.6)	0.661
	No	125 (21.0)	57 (22.4)	

Infection	Yes	217 (36.5)	96 (37.6)	0.745
	No	378 (63.5)	159 (62.4)	

Organ dysfunction	Yes	248 (41.7)	101 (39.6)	0.573
	No	347 (58.3)	154 (60.4)	

Vent	Yes	307 (51.6)	133 (52.2)	0.881
	No	288 (48.4)	122 (47.8)	

Comorbidities	Diabetes	133 (22.4)	57 (22.4)	0.195
	Hypertension	311 (52.3)	142 (55.7)	0.244
	Liver disease	140 (23.5)	58 (22.7)	0.255
	Neurological	81 (13.6)	28 (11.0)	0.279
	Renal failure	87 (14.6)	33 (12.9)	0.233
	Coagulopathy	120 (20.2)	62 (24.3)	0.252
	Alcohol abuse	140 (23.5)	65 (25.5)	0.240
	Fluid electrolyte	282 (47.4)	135 (52.9)	0.253
	Chronic pulmonary	102 (17.1)	35 (13.7)	0.267
	Cardiac arrhythmias	179 (30.1)	85 (33.3)	0.318
	CHF	145 (24.4)	61 (23.9)	0.220

Scoring systems	SAPSII	37 (27–47)	35 (26–44)	0.208
	Elixhauser score	8 (4–15)	9 (2–15)	0.636

Laboratory events	PT	14.1 (12.9–16.0)	14.1 (13.0–16.1)	0.970
	PTT	29.5 (25.7–34.1)	28.9 (25.8–34.5)	0.930
	Bilirubin	0.9 (0.5–2.2)	0.8 (0.4–1.9)	0.174
	Chloride	103.0 (99.0–108.0)	103.0 (98.0–108.0)	0.368
	ALT	42.0 (23.0–138.0)	41.0 (19.0–104.0)	0.242
	AST	59 (28.0–146.0)	54.0 (27.0–129.0)	0.414
	Calcium	8.3 (7.6–8.9)	8.3 (7.7–9.0)	0.529
	Anion gap	16.0 (13.0–19.0)	16.0 (14.0–19.0)	0.419
	Bicarbonate	23.0 (19.0–26.0)	23.0 (20.0–26.0)	0.548
	Creatinine	1.1 (0.8–1.9)	1.1 (0.7–2.0)	0.745
	Glucose	135.0 (105.0–180.0)	125.0 (98.0–163.0)	0.012
	Potassium	4.1 (3.7–4.6)	4.1 (3.7–4.6)	0.777
	Sodium	138.0 (135.0–141.0)	138.0 (135.0–141.0)	0.990
	Hematocrit	35.2 (30.3–40.7)	35.1 (31.3–40.3)	0.819
	Hemoglobin	11.8 (10.1–13.7)	11.9 (10.3–13.7)	0.602
	Platelet	226.0 (158.0–320.0)	220.0 (156.0–292.0)	0.668
	RDW	14.5 (13.6–15.7)	14.5 (13.5–15.9)	0.428
	WBC	12.6 (8.5–17.6)	12.6 (8.8–17.4)	0.794

Vital signs	MBP	80 (73–90)	78 (71–89)	0.147
	SPO_2_	97 (95–98)	97 (95–99)	0.664
	Temperature	37.0 (36.6–37.6)	37.0 (36.6–37.6)	0.909
	Heart rate	96 (83–109)	94 (82–109)	0.050
	Respiratory rate	21 (17–24)	20 (18–24)	0.403

LOS: length of stay; Vent: mechanical ventilation; CHF: chronic heart failure; SAPSII: Simplified Acute Physiology Score II; RDW: red blood cell distribution width; WBC: white blood cell; MBP: mean blood pressure.

**Table 2:** Multivariate Cox regression analysis of AP based on first 24 h data in the training set.

Variables	Multivariate analysis		
	HR	95% CI	*P* value
Weight	0.987	0.979–0.995	<0.001
Gender			
Male	Reference	—	—
Female	0.745	0.553–1.003	0.052

Insurance			
Medicare	Reference	—	—
Private	0.451	0.324–0.629	<0.001
Medicaid	0.615	0.387–0.978	0.040
Government	0.194	0.061–0.616	0.005
Self-pay	1.841	0.564–6.011	0.312

Explicit sepsis			
No	Reference	—	—
Yes	2.060	1.470–2.886	<0.001
SAPSII	1.035	1.025–1.046	<0.001
Elixhauser score	1.022	1.007–1.038	0.004
Bilirubin	1.048	1.015–1.082	0.005
Anion gap	1.033	1.012–1.055	0.002
Creatinine	0.856	0.788–0.930	<0.001
Hematocrit	1.122	1.049–1.201	<0.001
Hemoglobin	0.660	0.539–0.808	<0.001
RDW	1.138	1.069–1.211	<0.001
SPO_2_	0.939	0.902–0.978	0.002
Respiratory rate	1.023	0.992–1.054	0.147

SAPSII: Simplified Acute Physiology Score II; RDW: red blood cell distribution width.

## Data Availability

The data are available at https://physionet.org/content/mimiciii-demo/1.4/.

## Abbreviations

SAP: Severe acute pancreatitis
MIMIC-III: Medical Information Mart for Intensive Care III
C-index: Harrell's concordance index
AUC: Area under the receiver operating characteristic curve
DCA: Decision-curve analysis
NRI: Net reclassification improvement
IDI: Integrated discrimination improvement
AP: Acute pancreatitis
MODS: Multiple organ dysfunction syndrome
ICUs: Intensive care units
IQR: Interquartile range
SAPSII: Simplified Acute Physiology Score II
LOS: Length of stay
Vent: Mechanical ventilation
CHF: Chronic heart failure
RDW: Red blood cell distribution width
WBC: White blood cell
MBP: Mean blood pressure.

## References

[1] Zhenhai Ye W. T. L. Y. (2008). Clinical observation of acute pancreatitis complicated with sepsis. *Ningxia Medical Journal*.

[2] Chen C. C., Wang S. S., Lee F. Y. (2007). Action of antiproteases on the inflammatory response in acute pancreatitis. *JOP*.

[3] Sekimoto M., Takada T., Kawarada Y. (2006). JPN Guidelines for the management of acute pancreatitis: epidemiology, etiology, natural history, and outcome predictors in acute pancreatitis. *Journal of Hepato-Biliary-Pancreatic Surgery*.

[4] Yang J., Du Q., Chang W. (2019). Efficacy analysis of hemofiltration combined with early volume resuscitation in the treatment of severe acute pancreatitis complicated with septic shock. *Chinese Journal of Forensic Medicine*.

[5] Bakes K., Haukoos J. S., Deakyne S. J. (2016). Effect of volume of fluid resuscitation on metabolic normalization in children presenting in diabetic ketoacidosis: a randomized controlled trial. *Journal of Emergency Medicine*.

[6] Zhou L., Lai-Liu S., Cheng W., Zhou L., Lai-Liu S. U., Cheng W. (2012). An analysis of risk factors for sepsis secondary to severe acute pancreatitis. *Journal of Practical Shock (Chinese and English)*.

[7] Li S., Hu X., Xu J. (2019). Increased body mass index linked to greater short- and long-term survival in sepsis patients: a retrospective analysis of a large clinical database. *International Journal of Infectious Diseases*.

[8] Yao S., Jiang X., Sun C., Zheng Z., Wang B., Wang T. (2018). External validation and improvement of LiFe score as a prediction tool in critically ill cirrhosis patients. *Hepatology Research*.

[9] Iasonos A., Schrag D., Raj G. V., Panageas K. S. (2008). How to build and interpret a nomogram for cancer prognosis. *Journal of Clinical Oncology*.

[10] Pan Z., You H., Bu Q. (2019). Development and validation of a nomogram for predicting cancer-specific survival in patients with Wilms’ tumor. *Journal of Cancer*.

[11] Liu R.-Z., Zhao Z.-R., Ng C. S. H. (2016). Statistical modelling for thoracic surgery using a nomogram based on logistic regression. *Journal of Thoracic Disease*.

[12] Johnson A. E. W., Pollard T. J., Shen L. (2016). MIMIC-III, a freely accessible critical care database. *Scientific Data*.

[13] Huang Y.-L., Badrick T., Hu Z.-D. (2017). Using freely accessible databases for laboratory medicine research: experience with MIMIC database. *Journal of Laboratory and Precision Medicine*.

[14] Saeed M., Villarroel M., Reisner A. T. (2011). Multiparameter Intelligent Monitoring in Intensive Care II: a public-access intensive care unit database^∗^. *Critical Care Medicine*.

[15] Yang J., Li Y., Liu Q. (2020). Brief introduction of medical database and data mining technology in big data era. *Journal of Evidence-Based Medicine*.

[16] Wu W.-T., Li Y.-J., Feng A.-Z. (2021). Data mining in clinical big data: the frequently used databases, steps, and methodological models. *Military Medical Research*.

[17] Cheng B., Li D., Gong Y., Ying B., Wang B. (2020). Serum anion gap predicts all-cause mortality in critically ill patients with acute kidney injury: analysis of the MIMIC-III database. *Disease Markers*.

[18] Liu Z., Meng Z., Li Y. (2019). Prognostic accuracy of the serum lactate level, the SOFA score and the qSOFA score for mortality among adults with Sepsis. *Scandinavian Journal of Trauma, Resuscitation and Emergency Medicine*.

[19] Singer M., Deutschman C. S., Seymour C. W. (2016). The third international consensus definitions for sepsis and septic shock (Sepsis-3). *Journal of the American Medical Association*.

[20] Le Gall J. R., Lemeshow S., Saulnier F. (1993). A new Simplified Acute Physiology Score (SAPS II) based on a European/North American multicenter study. *Journal of the American Medical Association: The Journal of the American Medical Association*.

[21] Van Walraven C., Austin P. C., Jennings A., Quan H., Forster A. J. (2009). A modification of the elixhauser comorbidity measures into a point system for hospital death using administrative data. *Medical Care*.

[22] Banks P. A., Freeman M. L. (2006). Practice guidelines in acute pancreatitis. *American Journal of Gastroenterology*.

[23] Wu B. U., Johannes R. S., Sun X., Tabak Y., Conwell D. L., Banks P. A. (2008). The early prediction of mortality in acute pancreatitis: a large population-based study. *Gut*.

[24] Quan L., Zhang L., Lu H. (2009). The clinical significance of serum procalcitonin in differential diagnosis of acute pancreatitis with bacterial infectious systemic inflammatory syndrome. *Journal of Baotou Medical College*.

[25] Ranson J. H. C., Pasternack B. S. (1977). Statistical methods for quantifying the severity of clinical acute pancreatitis. *Journal of Surgical Research*.

[26] Di M.-Y., Liu H., Yang Z.-Y., Bonis P. A. L., Tang J.-L., Lau J. (2016). Prediction models of mortality in acute pancreatitis in adults. *Annals of Internal Medicine*.

[27] Larvin M., McMahon M. (1989). Apache-II score for assessment and monitoring of acute pancreatitis. *The Lancet*.

[28] Kumaravel A., Stevens T., Papachristou G. I. (2015). A model to predict the severity of acute pancreatitis based on serum level of amylase and Body Mass Index. *Clinical Gastroenterology and Hepatology*.

[29] Yang C. J., Chen J., Phillips A. R. J., Windsor J. A., Petrov M. S. (2014). Predictors of severe and critical acute pancreatitis: a systematic review. *Digestive and Liver Disease*.

[30] Gao W., Liu H. (2012). The value of serum procalcitonin in the diagnosis of early acute pancreatitis. *Chinese Journal of Experimental Diagnostics*.

[31] Martínez J., Sánchez-Payá J., Palazón J. M., Suazo-Barahona J., Robles-Díaz G., Pérez-Mateo M. (2004). Is obesity a risk factor in acute pancreatitis? A meta-analysis. *Pancreatology*.

[32] Segersvard R., Sylvan M., Herrington M., Larsson J., Permert J. (200l). Obesity increases the severity of acute experimental pancreatitis in the rat. *Scandinavian Journal of Gastroenterology*.

[33] Hong S., Qiwen B., Ying J., Wei A., Chaoyang T. (2011). Body mass index and the risk and prognosis of acute pancreatitis. *European Journal of Gastroenterology and Hepatology*.

[34] Sadr-Azodi O., Orsini N., Andrén-Sandberg Å., Wolk A. (2013). Abdominal and total adiposity and the risk of acute pancreatitis: a population-based prospective cohort study. *American Journal of Gastroenterology*.

[35] Yadav D., Lowenfels A. B. (2013). The epidemiology of pancreatitis and pancreatic cancer. *Gastroenterology*.

[36] Popa C. C., Badiu D. C., Rusu O. C., Grigorean V. T., Neagu S. I., Strugaru C. R. (2016). Mortality prognostic factors in acute pancreatitis. *Journal of Medicine and Life*.

[37] Talamini G., Uomo G., Pezzilli R. (1999). Serum creatinine and chest radiographs in the early assessm ent of actue pancreatiti. *American Journal of Surgery*.

[38] Bin Z. (2016). Correlation between serum creatinine level and prognosis of patients with early acute severe pancreatitis. *Modern Digestion and Interventional Therapy*.

[39] Chaochao T., Yalan Z., Ying H. (2014). Clinical value of serum biochemical indexes in the diagnosis and treatment of biliary severe acute pancreatitis. *Practical Preventive Medicine*.

[40] Koutroumpakis E., Wu B. U., Bakker O. J. (2015). Admission hematocrit and rise in blood urea nitrogen at 24 h outperform other laboratory markers in predicting persistent organ failure and pancreatic necrosis in acute pancreatitis: a post hoc analysis of three large prospective databases. *American Journal of Gastroenterology*.

[41] Yang S. W., Zhou Y. J., Zhao Y. X. (2017). The serum anion gap is associated with disease severity and all-cause mortality in coronary artery disease. *Journal of Geriatric Cardiology*.

[42] Banerjee T., Crews D. C., Wesson D. E. (2019). Elevated serum anion gap in adults with moderate chronic kidney disease increases risk for progression to end-stage renal disease. *American Journal of Physiology—Renal Physiology*.

[43] Al-Jaghbeer M., Kellum J. A. (2015). Acid-base disturbances in intensive care patients: etiology, pathophysiology and treatment. *Nephrology Dialysis Transplantation*.

[44] Jiang X., Su Z., Wang Y. (2019). Prognostic nomogram for acute pancreatitis patients: an analysis of publicly electronic healthcare records in intensive care unit. *Journal of Critical Care*.

[45] Goyal H., Awad H., Hu Z.-D. (2017). Prognostic value of admission red blood cell distribution width in acute pancreatitis: a systematic review. *Annals of Translational Medicine*.

[46] Han D. D., Shuo F. X., Zhuo C. L. (2020). A Novel Nomogram for predicting survival in patients with severe acute pancreatitis: an analysis based on the large MIMIC-III Clinical Database. *Preprint*.

